# Medication-Related Problems and Their Intervention in the Geriatric Population: A Review of the Literature

**DOI:** 10.7759/cureus.44594

**Published:** 2023-09-02

**Authors:** Alaa H Falemban

**Affiliations:** 1 Department of Pharmacology and Toxicology, Umm Al-Qura University, Makkah, SAU

**Keywords:** saudi arabia, stopp/start criteria, beers criteria, geriatrics, medication-related problems, inappropriate medication, elderly, polypharmacy

## Abstract

In order to implement the principles of providing clinically and economically effective care, the current state of healthcare must be evaluated, and challenges must be addressed. As part of a physician’s role in such a context, one tool consists of identifying medication-related problems (MRPs) and accordingly implementing best practices and innovative strategies to improve patient healthcare outcomes. The geriatric population is expected to have passed through the natural ageing process and experienced several physiological and biological changes that impact their bodies and lives. In the presence of geriatric syndromes and the increased number of medications consumed, the risk of MRPs such as polypharmacy, potentially inappropriate medication (PIM), adverse events, drug-drug interactions, and risk of non-adherence increases. Different interventions that focus on practical and perceptual barriers have been studied, and different tools to define clinically important prescribing problems relating to PIM have been established. The Beers Criteria and STOPP (Screening Tool of Older Persons' Prescriptions)/START (Screening Tool to Alert to Right Treatment) criteria are the most widely used sets of explicit PIM criteria; however, they are still limited in Saudi Arabia. These tools should be considered in clinical settings to improve healthcare outcomes in the geriatric population, and the clinical relevance of enhancing medication should also be explored from the point of view of both the patient and healthcare practitioners.

## Introduction and background

Healthcare is a primary focus of Saudi Vision 2030, and the National Transformation Program 2020 (NTP) aims to improve the quality of health services and amenities throughout the kingdom. Reducing ineffective care, carefully evaluating treatments clinically and economically, and adopting an active prevention strategy rather than a purely curative approach are all among the principles that have been taken into account when designing the model of care in line with Saudi Vision 2030 [1]. However, to achieve these principles, the current state of healthcare must be evaluated, and challenges must be addressed. As part of a clinician’s role in such a context, one of the tools consists of identifying medication-related problems (MRPs) and accordingly implementing best practices and innovative strategies to improve patient outcomes and, ultimately, population health [2].

MRPs are defined as events or circumstances related to a patient’s medication therapy that potentially or actually interfere with desired health outcomes [3,4]. Most studies on this subject are concerned with "medication error", which, by definition, refers to errors in prescribing or delivering medication to a patient rather than ill effects experienced by the patient following the administration of the medication. By contrast, MRP is a more comprehensive/inclusive term that covers not only medication error but other issues as well such as inappropriate medication, inappropriate dose, therapeutic failure, or failure to receive medication [5]. In any event, MRPs are associated with increase in morbidity and mortality, which leads to the overutilization of healthcare resources and, consequently, increased costs for the healthcare system [5]. A number of studies have shown that drug use accounts for 6-7% of all hospital admissions and that at least half of these admissions can be prevented [6]. In a recent meta-analysis summarizing studies conducted at healthcare facilities in Saudi Arabia, it was seen that the prevalence of drug-related problems linked to emergency departments and intensive care units was 16% [7]. Of these, commonly implicated cases included adverse drug reactions, medication non-adherence, drug overdoses, and drug interactions, while the most frequently used drugs involved were those that affect the central nervous systems and cardiovascular systems [7]. As noted by other studies, the majority of such cases are preventable, accounting for up to 80% of the reported drug-related problems [8]. However, although this preventability is distressing, it also indicates the possibility of interventions to reduce MRPs, hospitalizations, and deaths related to drug use.

Indeed, clinicians have developed interventions to address these issues; for example, active reporting for an adverse drug effect or using a drug interaction checker to ensure the safety of certain drugs co-administered. Medication reviews, also known as medication reassessments, are another intervention that has been shown to enhance medication safety and effectiveness, as well as reduce the incidence of MRPs [8].

However, these interventions are often conducted in inpatient settings where the accessibility, availability, and quality of resources are more convenient than those provided in outpatient settings [9]. Furthermore, in contrast to inpatient settings, the outpatient himself is suggested to be the one responsible for monitoring the entire medication regimen. It is common for such patients to be distracted and overwhelmed with information and thus pay less attention to medication instructions that often are not always tailored to their literacy levels or informational needs, which makes them more vulnerable to MRPs [10].

Among the other things noted regarding interventions is that most are carried out by the clinician without paying much attention to the patient. It is essential to understand how patients experience medicine and whether these interventions are aligned with their actual experience [4].

## Review

MRPs in the geriatric population

Patients’ categories and their medical situations are other essential points that need to be taken into account with regard to MRPs and subsequent interventions. Geriatric or elderly patients, patients with chronic kidney or liver diseases, patients in intensive care units, and paediatric patients are among the categories discussed with concern regarding MRPs. The elderly population is the group that is expected to be most affected by MRPs [11]. The older population is expected to have passed through the natural ageing process and experienced several physiological and biological changes that impact their bodies and lives. Frailty, functional limitation, falls, depression, cognitive impairment, incontinence, and malnutrition are among the most common health problems that interfere with daily living. Because these conditions do not fit into a specific category, they are referred to as geriatric syndromes [12]. In the presence of geriatric syndromes and the increased number of medications consumed, the risk of MRPs including polypharmacy, inappropriate medication, adverse events, risk of drug-drug interactions (DDIs), and risk of non-adherence increases. There is a cycle where older frail adults develop higher levels of MRPs, which in turn contributes to further frailty development [13].

Polypharmacy

Polypharmacy is a big concern when talking about MRPs in the geriatric population. The term "polypharmacy" refers to a patient's use of multiple medications. There may be two or more [14], four or more [15], or five or more distinct medications [16]. When more than 10 medications are used, the term "hyper-polypharmacy" is used [17]. In a recently published study, the prevalence of polypharmacy in a cohort in Saudi Arabia was seen to be 97.2% [18]. The prevalence of hyper-polypharmacy is 1% in the United States, 2.1% in New Zealand, 8% in Australia, 18% in Sweden, 28% in Finland, 31% in India, and (the highest) 40% in Saudi Arabia [17,18].

Alternatively, polypharmacy may also refer to using two or more medications for 240 days or longer [19], a daily use of multiple prescriptions including high-risk medications, or an individual’s non-essential use of multiple medications [20]. However, it has been suggested in a systematic review assessing the definition of polypharmacy that it may be necessary to replace numerical definitions with the term "appropriate polypharmacy" [21]. Such a definition could improve the assessment of multiple medication use in a comorbidity context [21]. If multiple medications are administered inappropriately, the term "polypragmasy" is used [22].

Regardless of the term's precise definition, unintended negative side effects caused by the use of multiple medications pose a significant health threat. With the increasing number of medications consumed, the risk of unwanted side effects and DDIs increases exponentially [23,24]. Furthermore, most adverse drug reactions due to polypharmacy can be confused with symptoms of geriatric syndrome or considered as a newly developed disease. Hence, “prescribing cascades”, where a new medication is added to the previously prescribed medicine intending to cure the illness, is a potential consequence of polypharmacy, resulting in even greater adverse effects [25]. In addition, there is a possibility that polypharmacy could lead to prescribing cascades where a new medication is added to the previous one intended to cure the illness.

However, not all polypharmacy is considered inappropriate. There is suitable and appropriate polypharmacy, and then there is non-suitable and inappropriate polypharmacy, and this must be closely examined during the process of establishing therapeutic plans for elderly patients [18]. Therefore, it is essential for clinicians to implement such considerations within their routine daily practices, having targets for medication use and a proposed stop point for any newly prescribed medication, setting up a clear plan for the end result of their medication use, and regular medication review [26]. Using such a practice would most likely reduce the number of drugs used by elderly patients and confirm the medications prescribed are actually necessary, not harmful or insufficient [18].

Non-adherence

Furthermore, due to the multiple medications often taken by elderly patients, non-adherence is a well-documented and expected common MRP in the geriatric population [27]. Indeed, not taking medication is not only a result of medications being overwhelming. Confusion, forgetting to take medications related to cognitive decline or because of a decline in physical function with ageing such as poor dexterity and impaired hearing are other issues that are concerned with non-adherence [28,29]. Moreover, complicated drug regimens should not be ignored. It has been shown that patients with complicated drug regimens have a seven-fold greater chance of not adhering to their medication [29]. Clearly, this finding emphasizes the importance of simplifying dose variations and reducing the number of prescribing frequencies. Nevertheless, according to a study conducted in Kuwait, non-adherence to long-term medications is not directly related to the number of medications and frequency at which they are taken, but rather to medicine being oral solid, non-oral formulations, prescription fees, and the need for support in using medications [30]. This suggests the need for more efforts to enhance communication between patients and healthcare professionals about medicines, practical difficulties in the experience of using medicines, and the perceived effectiveness of medicines [30].

A systemic review of the barriers affecting medication adherence in elderly patients identified a total of 80 factors in five categories to be associated with medication adherence in these adults. The five categories include patient factors, medication factors, physician factors, system-based factors, and other factors, all affecting poor medication adherence in elderly patients. However, the magnitude of each factor for adherence in the geriatric population could not be established [31]. Different interventions focusing on practical and perceptual barriers have been studied to improve adherence. Dual pharmaceutical intervention, medication review, and improvement of discharge letters [32], interventions on practical barriers, dosing aids such as multidose drug dispensing systems [33], extensive medication review, and better collaboration between healthcare providers and patients [8] are among attempts to improve adherence but with suboptimal outcomes.

Inappropriate medications

With ageing, the organs of the body also age. Several physiological and biological changes impact their functions and their dealing with medications, particularly with the two main pharmacokinetic-controlling body organs, the liver and kidney. Decreased liver function affects drug metabolism, and decreased renal function affects drug elimination [34]. In studies investigating Australian and American community-dwelling older persons and nursing home residents, 6-28% had been prescribed drugs that were either contraindicated or required dosage adjustments due to their kidney function [35]. In another study, rates of non-adherence to renal dosing guidelines ranged from 19% to 70% across all settings, with the highest rate occurring in outpatient care [36,34]. In several studies of drug use in hospitalized adults, 15-67% of drugs that require renal adjustment were given at inappropriate doses or intervals [37,38].

Apart from pharmacokinetic changes, the distinct changes in endogenous neurotransmitter concentrations within the nervous system make elderly people more prone to MRPs. Pharmacodynamic changes make elders more sensitive to the CNS effects of most drugs, especially if they are cognitively impaired. Therefore, inappropriate medications increase the risk of DDIs, complications of routine procedures, hospitalizations, and death [39]. 

Accordingly, a group of medications termed “potentially inappropriate medications” (PIMs) has been considered best avoided by elderly patients. The adverse risk of these medications exceeds their health benefits when prescribed in elderly patients and should typically be avoided in certain diseases or conditions.

In a multicentre study analysing PIMs in Chinese hospitalized patients with chronic disease and polypharmacy, the prevalence of PIM was 93.8%, with the majority related to insufficient doses and unnecessary medication therapy [40]. Hyperpolypharmacy (taking ≥10 medications) and the presence of coronary heart disease were the main factors of PIM [40]. Studies from Europe and North America indicate that PIMs and subsequent adverse drug reactions may cause 1.5-15% of emergency department visits and unplanned hospitalizations among older people [41]. In two cross-sectional retrospective studies conducted in the central region of Saudi Arabia, almost one out of two older adult patients (age ≥ 65 years) treated in an ambulatory care setting had used PIMs. The most commonly prescribed PIMs were gastrointestinal agents and endocrine agents. A high risk of PIM use was observed among those with ischemic heart disease, anxiety and polypharmacy [42,43].

Common interventions

The two Saudi studies investigating the incidences and factors influencing PIM use were based on the American Geriatric Society (AGS) Beers Criteria® [42,43]. In 1991, Mark Beers and colleagues created a list of drugs that were considered inappropriate for use in elderly patients [44]. With the support of the AGS and an expert panel, the criteria have been regularly updated about every three years since 2012. The 2019 AGS Beers Criteria included 30 individual criteria of medications or medication classes to be avoided in elderly patients and 16 criteria specific to more than 40 medications or medication classes that should be used with caution or avoided in certain diseases or conditions [44]. Table 1 shows an example of a drug in each category, recommendation, rationale, quality of evidence (QE) and strength of recommendation (SR) as given by the AGS. The intention of the AGS Beers Criteria is to improve medication selection, educate clinicians and patients, reduce adverse drug events, make drug dose adjustments based on kidney function, and serve as a tool for evaluating the quality of care, cost, and patterns of drug use of elderly patients [44].

**Table 1:** Sample of Beer’s Criteria Adapted from: American Geriatrics Society, 2019 [44], with permission from the publisher. QE: Quality of Evidence; SR: Strength of Recommendation; VTE: venous thromboembolism; NSAIDs: non-steroidal anti-inflammatory drugs

2019 American Geriatrics Society Beers Criteria® for:															
Potentially Inappropriate Medication Use in Older Adults															
System, Drug(s)		Rationale											Recommendation	QE	SR
Anticholinergics															
First-generation antihistamines:		Highly anticholinergic; clearance reduced with advanced age, risk of confusion. Use of diphenhydramine in situations such as acute treatment of severe allergic reaction may be appropriate											Avoid	Moderate	Strong
Brompheniramine Carbinoxamine Chlorpheniramine															
Anti-infective															
Nitrofurantoin		Potential for pulmonary toxicity, hepatoxicity, and peripheral neuropathy, especially with long-term use; safer alternatives available											Avoid in individuals with creatinine clearance	Low	Strong
Inappropriate Medication Use in Older Adults Due to Drug-Disease or Drug-Syndrome Interactions That May Exacerbate the Disease or Syndrome															
Disease/Syndrome				Drug(s)				Rationale				Recommendation		QE	SR
Gastrointestinal															
History of gastric or duoden al ulcer				Aspirin >325 mg/day Non –COX-2 –selective NSAIDs				May exacerbate existing ulcers or cause new/additional ulcers				Avoid unless other alternatives are not effective and patient can take gastroprotective agent (ie, proton-pump inhibitor or misoprostol		Moderate	Strong
Kidney/urinary tract															
Chronic kidney disease stage 4 or higher (creatinine clearance				NSAIDs (non-COX and COX selective, oral and parenteral, nonacetylated salicylates)				May increase risk of acute kidney injury and further decline of renal function				Avoid		Moderate	Strong
Potentially Inappropriate Medications: Drugs To Be Used With Caution in Older Adults															
Drug(s)			Rationale								Recommendation			QE	SR
Dabigatran Rivaroxaban			Increased risk of gastrointestinal bleeding compared with warfarin and reported rates with other direct oral anticoagulants when used for long-term treatment of VTE or atrial fibrillation in adults ≥75 years								Use with caution for treatment of VTE or atrial fibrillation in adults ≥75 years			Moderate	Strong
Potentially Clinically Important Drug-Drug Interactions That Should Be Avoided in Older Adults															
Drug	Interacting Drug					Risk Rationale			Recommendation					QE	SR
Lithium	Loop diuretics					Increased risk of lithium toxicity			Avoid; monitor lithium concentrations					Moderate	Strong
Warfarin	Amiodaron					Increased risk of bleeding			Avoid when possible; if used together, monitor INR closely					Moderate	Strong
Medications That Should Be Avoided or Have Their Dosage Reduced With Varying Levels of Kidney Function in Older Adults															
Medication					CrCl		Rationale			Recommendation				QE	SR
Cardiovascular or hemostasis															
Amiloride					<30		Increased potassium and decreased sodium			Avoid				Moderate	Strong
Apixaban					<25		Lack of evidence for efficacy and safety in patients with a CrCl			Avoid				Moderate	Strong

Another screening tool that attempts to define clinically important prescribing problems relating to PIMs is the STOPP (Screening Tool of Older Persons' Prescriptions)/START (Screening Tool to Alert to Right Treatment) criteria. These provide a screening tool for PIMs in elderly patients and for alerting doctors to the right treatment, which includes medications that should be considered the first-line choice to be prescribed for elderly patients. The STOPP/START criteria were reported to be more sensitive and to identify more medications associated with adverse drug events than the Beers criteria [45]. They are evidence-based physiological organized sets that were first launched by geriatricians from Cork University Hospital in Ireland in 2008. The latest version (version 3) was published in 2023 and consists of 190 criteria (133 STOPP and 57 START criteria) (Table 2) [46].

**Table 2:** Sample of STOPP/START Criteria Adapted from: O'Mahony et al., 2023 [46] STOPP: Screening Tool of Older Persons' Prescriptions; START: Screening Tool to Alert to Right Treatment; NYHA: New York Heart Association; SNRI: serotonin/noradrenaline reuptake inhibitors; SSRI: selective serotonin reuptake inhibitors

STOPP The following prescriptions are potentially inappropriate to use in patients aged 65 years and older.
Cardiovascular System
Digoxin for heart failure with normal systolic ventricular function (no clear evidence of benefit) Verapamil or diltiazem with NYHA Class III or IV heart failure (may worsen heart failure with reduced ejection fraction (HFREF)). Beta-blocker in combination with verapamil or diltiazem (risk of heart block).
Coagulation System
Long-term aspirin at doses greater than 100mg per day (increased risk of bleeding, no evidence for increased efficacy) Antiplatelet agents, vitamin K antagonists, direct thrombin inhibitors or factor Xa inhibitors with concurrent significant risk of major bleeding. Aspirin plus clopidogrel as long-term secondary stroke prevention i.e., > 4 weeks, (no evidence of added long-term benefit over clopidogrel monotherapy).
Central Nervous System
TriCyclic Antidepressants (TCAs) in patients with dementia, narrow angle glaucoma, cardiac conduction abnormalities, prostatism, chronic constipation, recent falls, prior history of urinary retention or orthostatic hypotension (risk of worsening these conditions). Initiation of TCAs as first-line treatment for major depression (higher risk of adverse drug reactions with TCAs than with SSRIs or SNRIs). SNRI, e.g., venlafaxine, duloxetine) and severe hypertension i.e., systolic blood pressure > 180 mmHg +/- diastolic blood pressure > 105 mmHg (likely to make hypertension worse).
START The following prescriptions are potentially inappropriate to use in patients aged 65 years and older.
Cardiovascular System
Statin therapy with a documented history of coronary, cerebral or peripheral vascular disease, unless the patient’s status is end-of-life or established moderate or severe frailty. Angiotensin Converting Enzyme (ACE) inhibitor with coronary artery disease. Beta-blocker with symptomatic coronary artery disease
Coagulation System
Vitamin K antagonists or direct thrombin inhibitors or factor Xa inhibitors in the presence of chronic or paroxysmal atrial fibrillation. Antiplatelet therapy (aspirin or clopidogrel or prasugrel or ticagrelor) with a documented history of coronary, cerebral or peripheral vascular disease.
Central Nervous System
L-DOPA or a dopamine agonist in idiopathic Parkinson’s disease with functional impairment and resultant disability. Non-TCA antidepressant for major depression. Acetylcholinesterase inhibitor (donepezil, rivastigmine, galantamine) for mild-moderate Alzheimer’s dementia.

In a systematic review exploring the practice of using explicit tools to review PIM in hospitalized patients, Beers criteria and STOPP/START criteria were found to be the most widely used sets of explicit PIM criteria. The hospital pharmacist initiates the intervention, detecting PIMs and reporting them to the attending physician or to a team of geriatricians and clinical pharmacists. The reduction of PIM varied in the studies, ranging from 3.5% up to 87%, with benzodiazepines being the most commonly detected PIM, followed by antipsychotics. Other common PIMs included proton pump inhibitors, digoxin, non-steroidal anti-inflammatory drugs (NSAIDs), and anticholinergics. Although not statistically significant, reductions in falls, hospitalization, and readmission have been reported after the reduction of PIM [47]. Regarding cost-effectiveness, the review indicated that one study reported approximately £63,000-144,000 in annual medication savings from a clinical PIM intervention [47].

In a study in Saudi Arabia that analysed PIM in 375 geriatric cardiology patients using AGS Beers Criteria 2012 and STOPP/START criteria, 44% and 47% were identified, respectively [48]. The most common PIMs according to the Beers Criteria were taking long-acting oral hypoglycaemic drugs and prolonged use of antihistamines and antipsychotic medications without proper indication. The most common PIM documented using STOPP/START criteria was related to overuse or underuse of proton pump inhibitors [48].

Indeed, these screening tools play an increasingly important part in both routine clinical practice and research relating to PIM [48].

Nevertheless, these tools don’t replace clinical judgement in individual cases and should be adapted as a guide for identifying medications for which the risks of use in elderly patients outweigh the benefits. They should be used in a complementary manner with shared clinical judgement regarding individual patient values and needs in making decisions about safe medication use in elderly patients. For a clinician who cannot find an alternative to a drug on this list, the criteria can serve as a reminder that close monitoring may be required as a consequence of the designation of the medication as potentially inappropriate. This will allow the possibility of adverse drug effects to be incorporated into the medical record and prevented or detected sooner.

In a recent cross-sectional, self-administered survey conducted in Jeddah, Saudi Arabia, 14.7% of pharmacists from seven hospitals and 10 community pharmacies used screening tools such as the STOPP and Beers Criteria to detect PIMs in elderly patients [49]. The authors state that PIM detection tools are not widely used in Saudi Arabia. The development of educational programmes to improve understanding of PIMs and facilitate the use of PIM screening tools in daily practices is therefore essential.

## Conclusions

In light of the above, MRPs including polypharmacy, non-adherence, inappropriate medication, adverse events, and DDIs in the elderly should be acknowledged as a key public health issue. Evaluating the current state of MRPs in elderly patients and addressing risk factors and challenges provides a starting point for preventing MRPs. Some evaluations conducted in Saudi Arabia indicate limited use of PIM detection tools. Implementing the use of such implicit or explicit tools accompanied by educational programs that help better understand these tools in daily practices, should be considered. The clinical relevance of enhanced medication use, from the perspective of both patients and healthcare practitioners, should also be explored.
